# Lifestyle counselling by persuasive information and communications technology reduces prevalence of metabolic syndrome in a dose–response manner: a randomized clinical trial (PrevMetSyn)

**DOI:** 10.1080/07853890.2020.1783455

**Published:** 2020-07-30

**Authors:** Young-Gyun Seo, Tuire Salonurmi, Terhi Jokelainen, Pasi Karppinen, Anna-Maria Teeriniemi, Junhee Han, Kyung Hee Park, Harri Oinas-Kukkonen, Markku J. Savolainen

**Affiliations:** a Department of Family Medicine, Hallym University Sacred Heart Hospital, Anyang, Gyeonggi-do, Republic of Korea; b Biocenter Oulu, Research Unit of Internal Medicine, University of Oulu, Oulu, Finland; c Research Center of Internal Medicine, Oulu University Hospital, Oulu, Finland; d Medical Research Center Oulu, Oulu University Hospital, University of Oulu, Oulu, Finland; e Unit of Medicine, Oulu University Hospital, Oulu, Finland; f Oulu Advanced Research on Service and Information Systems, Faculty of Information Technology and Electrical Engineering, University of Oulu, Oulu, Finland; g Department of Statistics, Hallym University, Chuncheon, Gangwon-do, Republic of Korea

**Keywords:** Metabolic syndrome, obesity, lifestyle, information and communication technology, behaviour change support system, persuasive systems design

## Abstract

**Objectives:**

The aim was to investigate whether lifestyle changes produced by persuasive Information and Communication Technology (ICT) counselling can lower the prevalence of metabolic syndrome (MetS).

**Methods:**

A total of 532 participants (20–60 years, body mass index 27–35 kg/m^2^) were randomly assigned to six arms according to counselling type (no, short-term, or intensive) with or without ICT intervention. In this report the prevalence of MetS and its components were compared between no-ICT group and ICT group. Moreover, the frequency of the web information system usage was analysed for the number of logins, responses to weekly messages, and other record variables.

**Results:**

The ICT group had significantly lower proportion of MetS (33.7% vs. 45.3%, *p* = .022) than the no-ICT group at 2-year follow-up. In mixed model, the ICT group had lower prevalence of MetS than no-ICT group (OR 0.50, 95%CI 0.27–0.90) after intervention. The tertile with the highest utilization had 71% lower prevalence of MetS compared with the lowest utilization tertile or the no-ICT group.

**Conclusions:**

Web-based ICT is able to reduce the prevalence of MetS. In addition, higher utilization of the web information system is associated with a greater decrease in the prevalence of MetS.

**Key messages**
Our internet health behaviour change support system based on persuasive design and cognitive behaviour therapy markedly reduces metabolic syndrome in overweight/obese subjects. As a stand-alone tool it may save healthcare personnel resources as it is suitable at a low cost for both obese/overweight patients and the public at large.

## Introduction

The worldwide prevalence of metabolic syndrome (MetS) has increased over the decades [1–3]. Individuals with MetS are more likely to develop occlusive vascular disease, coronary artery disease (CAD), diabetes, and stroke [4,5]. Furthermore, in a recent large population-based study MetS reduction was associated with lower risk for major adverse cardiovascular events than chronic MetS [6]. Moreover, MetS is associated not only with an increase in cardiovascular disease (CVD) but also with an increase in all-cause mortality [7]. In a recent longitudinal cohort study, both the severity and duration of obesity were positively associated with the incidence of MetS, suggesting that obesity without MetS is a transient state of the path to MetS [8]. Therefore, weight reduction should be recommended to all obese patients, including those without MetS.

However, MetS can be caused by metabolic derangement regardless of obesity. MetS without obesity is more dangerous than obesity without MetS. Recent studies have shown that MetS without obesity is associated with a higher prevalence of stroke [9], higher prevalence of CAD [10], and higher all-cause mortality compared with obesity without MetS [11]. Therefore, there is a need for an intervention method that can improve overall health by changing lifestyle as well as weight control.

Earlier observational (uncontrolled, non-randomized) studies suggested that lifestyle change programmes aiming at weight loss not only reduce weight but also positively affect the determinants and prevalence of MetS [12,13] and improve quality of life [14]. A systematic review has also shown that motivating MetS patients to maintain improved lifestyles is a key element in achieving a reduction in MetS components [15] and more recently, third-wave cognitive behaviour therapies (CBTs) including dialectical behavioural therapy, schema therapy, acceptance and commitment therapy, acceptance-based behavioural treatment, mindfulness-based cognitive behavioural treatment or compassion-focused therapy have been introduced [16].

The above-mentioned studies used lifestyle interventions for MetS without information and communication technology (ICT). Later, various ICT methods have been introduced. In a systematic review of lifestyle modification for MetS, it was stated that technologies such as mobile and internet-based communication can improve the lifestyle habits of MetS patients but are ineffective when compared with personal contact [15]. More recently, a web-based information system had positive effects on lifestyle (moderate physical activity (PA), walking and cholesterol intake) and quality of life [17]. In another internet-based lifestyle intervention, social media methods improved body weight, body mass index (BMI), waist circumference (WC), fat mass, lean mass, and energy intake [18].

In our PrevMetSyn (Prevention of Metabolic Syndrome) trial, the best weight loss results were obtained with the combination of CBT group counselling and ICT method, both with a specific emphasis on eating behaviour [19]. The present study investigates the results further by evaluating whether ICT counselling focussing on lifestyle changes can lower the prevalence of MetS. Internet log data of the ICT software was used in order to see if the frequency of the software usage affected the components of MetS in a dose-response manner.

## Materials and methods

### Trial design

The protocol of PrevMetSyn was a six-armed, parallel design, randomized clinical trial [19]. All procedures performed were in accordance with the 1964 Helsinki declaration and its later amendments or comparable ethical standards. Signed informed consent was obtained from all subjects before initiation of the intervention. The entire study protocol was approved by the Ethics Committee of the hospital district of Northern Ostrobothnia, Oulu, Finland (approval number 29/2012). This trial was registered in ClinicalTrials.gov (Identifier: NCT01959763).

### Participants

Using information from the Finnish Population Register Centre, letters of invitation to participate in the research were sent in a random manner to adults aged 20–60 years who lived in the city of Oulu. The inclusion criteria for those willing to participate were: BMI between 27 and 35 kg/m^2^, an internet-enabled environment, a health condition that is not a contraindication to weight reduction, and not current treatment for obesity. The exclusion criteria were uncontrolled current health factors such as abnormal laboratory values (thyroid, kidney and liver function tests) or clinically significant illness with contraindication for weight loss or PA. The prevalence of common chronic diseases (present or history) among the study subjects at the baseline was the following (number of subjects, and percentage in parenthesis): depression 44 (8.5%), asthma 45 (8.5%), joint diseases 16 (3.0%), coronary heart disease 9 (1.7%) and cancer 16 (3.0%).There were no differences between the No-ICT and ICT arms.

### Interventions

Supplementary Figure 1 shows the flow chart of the intervention. First, participants were divided into three groups that received intensive group counselling, short-term group counselling, or no group counselling. Each group was divided into two groups, one receiving and one not receiving ICT intervention. In this paper, the focus was ICT intervention (no-ICT vs. ICT).

Intensive group counselling aimed at 5% weight loss and maintenance, improved eating behaviour, and reduced risk of diabetes and CVD based on a cognitive-behavioural approach. The intensive group counselling consisted of 8–9 participants per group, conducted by two clinical nutritionists and students of clinical nutrition for 90 min each. Seven of the eight counselling sessions were held every two weeks, and the last counselling session was held one month later.

Short-term group counselling aimed not only at losing weight but also at change of lifestyle, including dietary habits. Short-term group counselling was conducted by two registered nurses for a total of two sessions, 90 min each. The usual care included informing participants of blood test results and providing information related to weight control and prevention of MetS. It is noteworthy that the study subjects in the ICT arm had the same number of visits to the study centre and no extra contacts with the study personnel compared with the subjects in the No-ICT arm. It is important to note that there were no intervention procedures between the 12-month and the 24-month visits.

The 52-week ICT intervention was designed and developed by using the Persuasive Systems Design methodology [20] for the PrevMetSyn study as a Health Behaviour Change Support System (HBCSS) [21], technically as a web-based information system based on a cognitive behavioural approach [22,23]. The main characteristics are weekly health-related contents, information on healthy lifestyle and self-monitoring related software functionalities. Therefore, the participants had a weekly contact with the web-based information system during the 12-month intervention.

The software functionalities included a variety of tasks, such as setting goals for body weight, exercise and eating habits, adopting healthy lifestyle choices, and overcoming risk situations for overeating. The self-monitoring section allowed participants to check the goals set and track the changes. In the diary, participants could write down how they felt during the intervention or about the process of change, and they could also make entries in the food diary.

Therefore, the ICT in this study is not a simple technology or means, but an independent intervention method. The web-based HBCSS was a stand‐alone ICT designed for the PrevMetSyn study. In addition to Persuasive Systems Design methodology and Cognitive Behavioural Therapy approach, current scientific and practical knowledge of eating behaviour, diet, PA and health information literacy has also been applied.

Body measurements and blood sampling were performed in the research laboratory of the Oulu University Hospital at screening visit and one and 2 years after the start of the intervention. Questionnaires were used to gather data on PA, tobacco use, alcohol use, sleep time, screen time (watch TV, DVDs or videos, and spend time on a computer or a game console during free time), eating while watching (eat while watching TV or spending time on a computer). The questionnaire data were collected during the visit or electronically by a personal internet link. In addition, 52 weeks of internet log data were collected in the ICT group. More detailed protocols can be found in our previously published study [19].

### Outcome

The primary outcome for this trial was body weight (or BMI). The secondary outcome measures were MetS and its components (WC, blood pressure (BP), fasting plasma glucose (FPG), high density lipoprotein (HDL), triglycerides (TG)), as well as total cholesterol (TC), low density lipoprotein (LDL), haemoglobin A1c (HbA1c) and plasma insulin.

The 2001 National Cholesterol Education Programme/Adult Treatment Panel III [24] and the 2005 American Heart Association/National Heart, Lung, and Blood Institute [25] criteria defined MetS as the presence of any three of the following five components: (1) WC ≥102 cm in men and ≥88 cm in women; (2) Serum TG ≥150 mg/dL or drug treatment for elevated TG; (3) Serum HDL <40 mg/dL in men and <50 mg/dL in women or drug treatment for low HDL; (4) BP ≥130/85 mmHg or drug treatment for elevated BP; (5) FPG ≥100 mg/dL or drug treatment for elevated blood glucose. This study followed this definition for MetS.

### Sample size and power

Sample size estimation based on 0.9 correlation between body weight measurements within individuals at 90% power with a two-sided significance level set at .05 showed that a minimum of 252 participants, 42 for each group, was required. In this paper, only the two groups (no-ICT vs. ICT) were used and the presence of MetS was used as the outcome variable. Therefore, the estimated power for a two-sample proportions test using the ratio of MetS (45.3%) in no-ICT group and the odds ratio of ICT group (0.50) in the mixed model is 97.0%.

### Randomization

An independent researcher used Microsoft Excel to generate a randomization list with 24 random permuted blocks. The randomization was done in two steps. First, participants were allocated to three groups that received intensive group counselling, short-term group counselling, or no group counselling. At the second stage, the participants were allocated to use or not to use the web-based ICT.

### Statistical analysis

The Shapiro–Wilk test was used to evaluate the normality of the data. Participants were divided into two groups according to whether they used the web-based ICT. General characteristics and MetS-related variables of the two groups at the 3 time points (baseline, 1 year, and 2 years) were compared using the two independent sample *t*-test or chi-squared test as appropriate for the type of data. Our primary analysis was to compare the prevalence of MetS between the two groups. We first analysed changes in the prevalence of MetS after internet-based lifestyle counselling using McNemar’s test. We used mixed effects logistic regression models to compare the prevalence of MetS across no-ICT group and ICT group, under the assumption that data were missing at random. Intercept was used to mixed model random effects at the individual level. Generalized estimating equations models with a logarithmic link function and an exchangeable covariance matrix were also used to evaluate the ICT intervention effects on the prevalence of MetS, under the assumption that data were missing completely at random. Mixed effects logistic regression and generalized estimating equations models were used to analyse the prevalence of MetS according to the utilization of the web information system using internet log data. In addition, mixed effect linear regression models were used for testing group by time interaction effects on MetS-related variables. All statistical analyses were conducted using Stata/MP, version 14.0 (StataCorp, College Station, TX, USA). All statistical tests were two-sided, and statistical significance was determined at a *p*-value of <.05.

## Results

### General characteristics of participants

A total of 532 participants aged 20–60 years and with BMI of 27–35 kg/m^2^ were enrolled. Supplementary Figure 2 shows the flow chart of the participants in the study. The 2-year follow-up rate was 70.5%. Supplementary Table 1 shows the general characteristics of study completers over time. There were no differences between ICT group and no-ICT group at baseline. In 2-year follow-up, ICT group had a slightly higher proportion of eating while watching than no-ICT group (85.4% vs. 76.1%, *p* = .049). There were no differences between no-ICT group and ICT group for the other variables at 1-year and 2-year follow-up.

The attrition rate was low, only 29.5% at the 2-year visit, and there was no difference in body weight (kg) between dropouts and completers at baseline (Total: dropouts 89.9 ± 10.8 vs. completers 89.3 ± 11.4, *p* = .555; No ICT group: dropouts 89.9 ± 11.7 vs. completers 89.6 ± 11.6, *p* = .842; ICT group: dropouts 90.0 ± 9.7 vs. completers 89.1 ± 11.3, *p* = .530). WC (cm) did not differ between dropouts and completers at baseline (Total: dropouts 101.6 ± 8.2 vs. completers 100.7 ± 7.7, *p* = .196; No ICT group: dropouts 101.1 ± 7.0 vs. completers 101.1 ± 7.9, *p* = .969; ICT group: dropouts 102.2 ± 9.4 vs. completers 100.2 ± 7.8, *p* = .073).

### Metabolic syndrome related variables of participants over time

Table 1 shows cardiometabolic risk markers of study participants over time. At baseline, there were no differences between ICT group and no-ICT group except for plasma insulin (12.0 ± 5.5 vs. 13.4 ± 6.6, *p* = .033). In 1-year follow-up, BMI (*p* = .001) and WC (*p* = .004) were significantly lower in ICT group than in no-ICT group. In addition, plasma insulin was significantly lower (*p* = .024) and QUICKI was significantly higher (*p* = .015) in ICT group than in no-ICT group. Similarly, BMI (*p* = .002) and WC (*p* = .008) were significantly lower in ICT group than in no-ICT group at 2-year follow-up. In addition, plasma insulin (*p* = .038) and HOMA-IR (*p* = .033) were significantly lower in ICT group than in no-ICT group.

**Table 1.** Cardiometabolic risk markers in no-ICT group and ICT group over time.

Variables	Baseline			1-year follow-up			2-year follow-up		
	No ICT (*n* = 179)	ICT (*n* = 196)	*p* Value	No ICT (*n* = 179)	ICT (*n* = 196)	*p* Value	No ICT (*n* = 179)	ICT (*n* = 196)	*p* Value
Age, year	46.5 ± 9.5	46.5 ± 9.3	.959						
Sex, *n* (%)			.733						
Men	90 (50.3)	102 (52.0)							
Women	89 (49.7)	94 (48.0)							
Clinical characteristics									
Body weight (kg)	89.6 ± 11.6	89.1 ± 11.3	.653	88.8 ± 11.8	86.8 ± 12.0	.103	89.1 ± 11.8	87.3 ± 12.2	.144
BMI (kg/m^2^)	30.4 ± 2.1	30.1 ± 2.0	.152	30.2 ± 2.5	29.3 ± 2.2	.001	30.3 ± 2.6	29.5 ± 2.3	.002
WC (cm)	101.1 ± 7.9	100.2 ± 7.6	.259	100.5 ± 8.3	97.9 ± 8.6	.004	99.9 ± 8.4	97.5 ± 8.9	.008
SBP (mmHg)	131.8 ± 17.7	129.8 ± 15.7	.246	125.1 ± 17.1	124.3 ± 16.9	.637	127.5 ± 17.3	124.6 ± 14.5	.077
DBP (mmHg)	83.1 ± 10.8	82.4 ± 10.1	.538	79.3 ± 10.3	78.3 ± 10.1	.360	80.9 ± 11.2	79.5 ± 9.7	.179
Glucose metabolism									
HbA1c (%)	5.66 ± 0.38	5.69 ± 0.48	.593	5.58 ± 0.35	5.65 ± 0.51	.097	5.49 ± 0.34	5.49 ± 0.48	.959
FPG (mmol/L)	5.51 ± 0.46	5.54 ± 0.63	.529	5.51 ± 0.48	5.49 ± 0.62	.696	5.51 ± 0.57	5.42 ± 0.66	.165
Insulin (mIU/L)	13.4 ± 6.6	12.0 ± 5.5	.033	12.9 ± 7.2	11.4 ± 5.9	.024	13.5 ± 7.8	12.0 ± 6.1	.038
HOMA-IR	3.33 ± 1.84	2.99 ± 1.49	.051	3.23 ± 2.00	2.86 ± 1.82	.063	3.40 ± 2.26	2.95 ± 1.78	.033
QUICKI	0.33 ± 0.02	0.33 ± 0.02	.114	0.33 ± 0.02	0.34 ± 0.03	.015	0.33 ± 0.03	0.33 ± 0.03	.091
Plasma lipids and lipoproteins									
TC (mmol/L)	5.33 ± 0.99	5.43 ± 0.99	.327	5.27 ± 0.99	5.33 ± 1.07	.610	5.16 ± 0.94	5.33 ± 1.01	.109
HDL (mmol/L)	1.45 ± 0.33	1.49 ± 0.40	.322	1.42 ± 0.31	1.48 ± 0.41	.103	1.45 ± 0.38	1.52 ± 0.44	.097
LDL (mmol/L)	3.60 ± 0.88	3.66 ± 1.01	.562	3.58 ± 0.94	3.47 ± 0.96	.278	3.52 ± 0.89	3.61 ± 0.97	.371
TG (mmol/L)*	1.30 ± 1.64	1.26 ± 1.67	.515	1.18 ± 1.62	1.15 ± 1.74	.726	1.21 ± 1.60	1.17 ± 1.61	.444

Data are presented as mean ± standard deviation for continuous variables (*T* test) and numbers (%) for categorical variables (χ^2^ test). Percentages have been rounded and may not total 100.

*Geometric mean ± standard deviation.

ICT: Information and Communication Technology; BMI: body mass index; WC: waist circumference; SBP: systolic blood pressure; DBP: diastolic blood pressure; HbA1c: haemoglobin A1c; FPG: fasting plasma glucose; TC: total cholesterol; HDL: high-density lipoprotein; LDL: low-density lipoprotein; TG: triglycerides; HOMA-IR: Homeostatic Model Assessment for Insulin Resistance (calculated according to the formula: fasting insulin (μU/mL) × fasting glucose (mmol/L)/22.5); QUICKI: Quantitative Insulin Sensitivity Check Index (derived using the inverse of the sum of the logarithms of fasting insulin and fasting glucose: 1 / (log(fasting insulin (μU/mL)) + log(fasting glucose(mmol/L)/0.0555)).

### Effects of ICT intervention on metabolic syndrome

**Figure 1.**
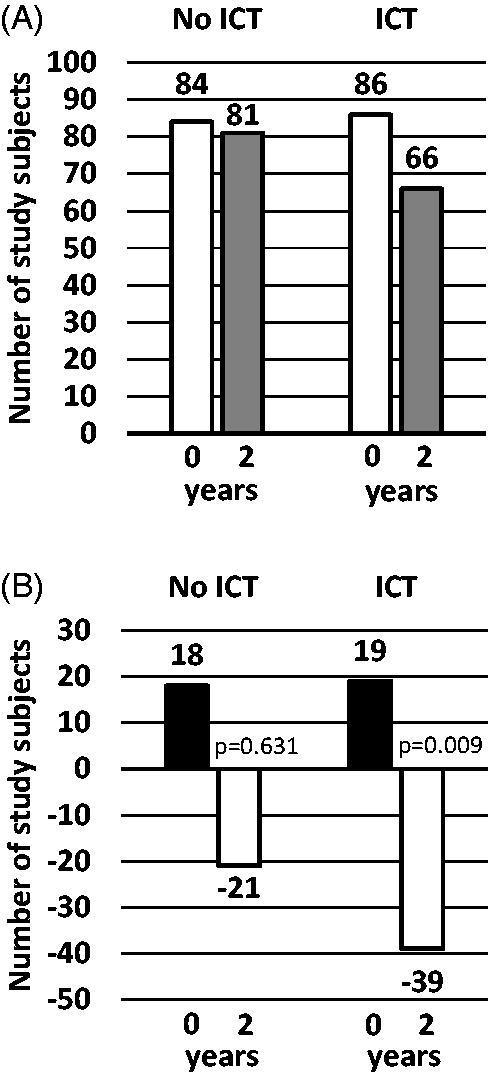
Changes in the prevalence of metabolic syndrome after internet-based lifestyle counselling (between baseline and 2 years). Panel A shows the number of subjects with metabolic syndrome at baseline (white bars) and at 2-year visit (grey bars). Black bars in Panel B show the number of subjects who developed metabolic syndrome from baseline to the 2-year visit while white bars show subjects who met the metabolic syndrome criteria at baseline but not at the 2-year visit. *p* values from McNemar’s Test. ICT: Information and Communication Technology.

ICT group had significantly lower proportion of MetS (33.7% vs. 45.3%, *p* = .022) than no-ICT group at 2-year follow-up (Table 2). The prevalence of MetS was reduced by 23% from 86 cases at baseline to 66 cases at the 2-year follow-up (*p* = .009) in the ICT group whereas no significant change was observed in the no-ICT group (84 subjects with MetS at baseline and 81 at the 2-year follow-up; Figure 1(A)). In the ICT group the number of improved cases of MetS was significantly higher than that of new cases of MetS (39 vs. 19, *p* = .009) while in the no-ICT group there was no difference between improved cases of MetS and new cases of MetS (21 vs. 18; Figure 1(B)).

**Table 2.** Prevalence of metabolic syndrome and its components in no-ICT group and ICT group among completers.

Variables	Baseline			1-year follow-up			2-year follow-up		
	No ICT (*n* = 179)	ICT (*n* = 196)	*p* Value	No ICT (*n* = 179)	ICT (*n* = 196)	*p* Value	No ICT (*n* = 179)	ICT (*n* = 196)	*p* Value
MetS prevalence, *n* (%)	84 (46.9)	86 (43.9)	.553	68 (38.2)	66 (33.7)	.362	81 (45.3)	66 (33.7)	.022
Components of MetS*									
MetS – WC	141 (78.8)	150 (76.5)	.603	127 (71.4)	130 (66.3)	.296	124 (69.3)	118 (60.2)	.067
MetS – TG	68 (38.0)	59 (30.1)	.107	52 (29.2)	41 (20.9)	.064	59 (33.0)	53 (27.0)	.211
MetS – HDL	50 (27.9)	52 (26.5)	.761	53 (29.8)	50 (25.5)	.356	63 (35.2)	55 (28.1)	.137
MetS – BP	119 (66.5)	116 (59.2)	.145	101 (56.7)	94 (48.0)	.090	109 (60.9)	100 (51.0)	.055
MetS – FPG	75 (41.9)	86 (43.9)	.699	68 (38.2)	80 (40.8)	.606	76 (42.5)	64 (32.7)	.050
Number of MetS components			.613			.172			.053
0	7 (3.9)	16 (8.2)		11 (6.2)	29 (14.8)		18 (10.1)	28 (14.3)	
1	31 (17.3)	37 (18.9)		49 (27.5)	48 (24.5)		30 (16.8)	55 (28.1)	
2	57 (31.8)	57 (29.1)		50 (28.1)	53 (27.0)		50 (27.9)	47 (24.0)	
3	41 (22.9)	43 (21.9)		34 (19.1)	34 (17.4)		39 (21.8)	33 (16.8)	
4	30 (16.8)	32 (16.3)		20 (11.2)	21 (10.7)		26 (14.5)	23 (11.7)	
5	13 (7.3)	11 (5.6)		14 (7.9)	11 (5.6)		16 (8.9)	10 (5.1)	

Data are presented as numbers of subjects (%) for categorical variables (χ^2^ test). Percentages have been rounded and may not total 100.

*See Materials and Methods section for the cut-off points of the criteria of the 2001 NCEP-ATP III and the 2005 AHA/NHLBI.

ICT: Information and Communication Technology; MetS: metabolic syndrome; WC: waist circumference; TG: triglycerides; HDL: high-density lipoprotein; BP: blood pressure; FPG: fasting plasma glucose.

We used mixed effects logistic regression and generalized estimation equations models for MetS to see if the difference between the two groups was maintained after adjusting the effects of other factors (Supplementary Table 2). We used age at baseline, sex, group, time, obesity, current tobacco use, eating while watching, and type of group counselling (no, short-term, or intensive) as explanatory variables. Intercept was used to mixed model random effects at the individual level.

Older age, obesity, current tobacco use and eating while watching were associated with a net increase in the prevalence of MetS, while female gender and ICT group were associated with a net decrease in the prevalence of MetS. The results were the same, when the sleep time and current PA were further adjusted, and mixed-effects logistic regression and generalized estimation models were performed.

### Effects of utilization of the web information system on metabolic syndrome

**Figure 2.**
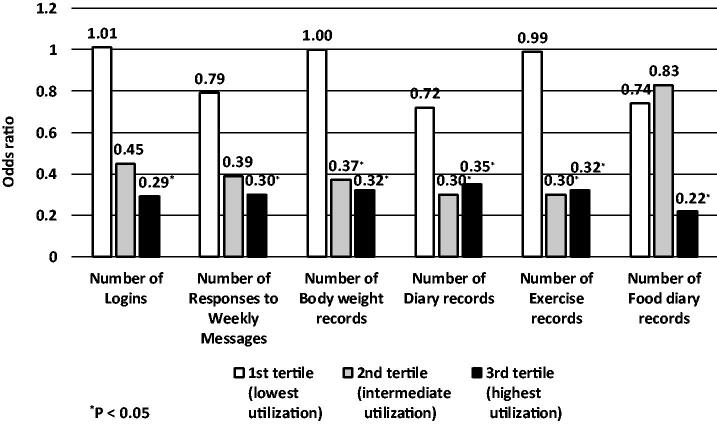
Effect of the utilization of the ICT software on the prevalence of metabolic syndrome after follow-up of 2 years. The ICT utilization was divided into tertiles (low, intermediate or high usage of the various aspects of the software). The no-ICT group was used as the reference in the mixed model analysis. ICT: Information and Communication Technology.

If there is a difference in the prevalence of MetS according to the degree of utilization of the web information system, the effect of ICT can be indirectly estimated. Therefore, we used mixed logistic regression and generalized estimation equations models to analyse the prevalence of MetS according to the utilization of the web information system using internet log data (Supplementary Table 3 and Figure 2). Each log data were categorized as tertile and analysed to see if there was a difference in the prevalence of MetS from the lowest utilization group to the higher utilization tertile compared with no-ICT group. There was no difference between the lowest utilization tertile and the no-ICT group in the prevalence of MetS in any of the variables describing the frequency of usage. By contrast, in the tertile with the highest utilization the number of logins, number of responses to weekly tasks and food diary records variables were associated with 71%, 70% and 78% lower prevalence of MetS, respectively, compared with the no-ICT group. For the number of body weight records, the number of diary records and the number of exercise records variables, the prevalence of MetS was 63%, 70% and 70% lower in the tertile with intermediate utilization, and 68%, 65% and 68% lower in the tertile with highest utilization, respectively compared with the no-ICT group.

### Effects of ICT intervention on metabolic syndrome related variables

The mixed effect linear regression models were used for testing group by time interaction effects on MetS-related variables after adjusting for age and sex (Supplementary Table 4). In 1-year follow-up, significant group by time interaction effects were found on body weight (*p* = .001), BMI (*p* < .001), WC (*p* < .001), and LDL (*p* = .01). In 2-year follow-up, significant group by time interaction effects were found on body weight (*p* = .005), BMI (*p* = .002), and WC (*p* = .001). No significant group by time interaction effects were found on the other MetS-related variables.

## Discussion

We have previously reported that the internet-based software with persuasive design features developed in our previous trial helped to reduce body weight in overweight and obese subjects compared with face-to-face group counselling or no counselling [19]. Here we investigated whether the ICT software can lower the prevalence of MetS and its components. We found a marked reduction in the prevalence of MetS. Moreover, the decrease was associated with the frequency by which the participants used the net-based health behaviour change support system (HBCSS), i.e. the usage of the software reduced the prevalence of MetS in a dose–response manner.

Previous studies have shown that obesity, MetS, cardiometabolic risk factors and even diabetes can be improved, reverted or cured by lifestyle changes by intensive counselling [26,27]. Moreover, the risk for major adverse cardiovascular events can be reduced by curing MetS [6]. Recently, a review by Lawlor *et al.* [16] summarized third-wave CBTs that may include dialectical behavioural therapy, schema therapy, acceptance and commitment therapy, acceptance-based behavioural treatment, mindfulness-based cognitive behavioural treatment or compassion-focused therapy. The ICT used here comprised some aspects of these CBT categories. Changes in eating behaviour are important for weight reduction [28]. Therefore, eating behaviour was the main focus of the HBCSS used here.

There are thousands of weight loss and management apps but only a few have been tested in rigorous scientific trials [29,30]. However, in most cases software features, user experiences or actual outcomes have not been clearly reported. By contrast, our previous report showed sustained beneficial effects of the internet-based HBCSS on body weight [19]. Moreover, the application has been systematically developed with a set of persuasive software features being carefully selected, designed and programmed into the application for improving patients’ behaviour. User experience studies of these persuasive features have been reported earlier [22,23].

Our internet-based software has all the features described above, i.e. focus on eating behaviour, counselling based on cognitive behavioural therapy, and internet-based weekly contact with a persuasive systems design. Here we show that the combined effect of these features was beneficial compared with no-ICT intervention, but the analysis carried out in this paper does not make it possible to pinpoint which one of these various components is the main effector.

Some studies have shown improved body composition and cardiometabolic risk through ICT intervention [29,31]. In most cases, however, only short-term (≤ 6 months) weight loss with the intervention-induced weight loss of small clinical significance has been studied. A recent review compared the effectiveness of web-based digital health interventions with offline interventions on weight loss and lifestyle habit changes [32]. The weight loss was followed up for more than six months in only in three studies. Here, the follow-up was 2 years.

For the number of logins, weekly task records, body weight records, diary records, exercise records, and food diary records variables, the prevalence of MetS was lower in the highest utilization group than in the no-ICT group (Supplementary Table 3 and Figure 2). Some ICT intervention studies have shown a dose–response relationship: the higher the utilization of ICT, the greater the impact on body composition or cardiometabolic risk factors including body weight, BMI, body fat percentage, and WC among active participants compared with less active participants in a mobile health care programme [33]. Another RCT reported that participants in an interactive web-based intervention who logged in five or more times during the intervention period improved lifestyle related to MetS [17].

Thus, the level of participation may be related to improved weight-related outcomes, thereby improving health outcomes. However, previous studies have not investigated the effect of actual system usage found in log data for each category as was done in our study.

The 2-year follow-up rate of this study was 70.5%, i.e. similar to other lifestyle interventions [26,34]. As previous ICT interventions for obesity or MetS had shorter follow-up periods and the follow-up rates have varied from 73.1% to 79.1% during the 6 months intervention [17,33,35], this study is fully feasible and generalizable, and long-term applicable.

### Strengths and limitations

The strength of this study is in the results of the 2-year follow-up through ICT intervention showing that MetS prevalence and MetS-related variables can be improved by ICT-counselling. We also observed a dose–response relationship between ICT intervention and MetS by showing that the higher the participation of ICT or system usage, the lower the MetS prevalence. All components of MetS tended to improve although the absolute changes in single components were relatively small, e.g. only about 2.5 cm in WC. However, the combined effect of all components was significant. Thus, the ICT intervention leads to a beneficial effect on MetS prevalence as reported here and also decreases the risk of type 2 diabetes as determined by the ceramide score in our previous report [36].

Importantly, the HBCSS to induce change of behaviour was used by the participants themselves, thus the support software was a stand-alone system requiring no assistance by healthcare professionals. By using HBCSS, it is expected that the long-term effects will be sustained even after the intervention is over by changing the participants’ perceptions [23]. This study showed that the effect remained at the 2-year visit, i.e. 1 year after the end of the intervention.

This study has some limitations. First, it was not possible to blind the participants in this kind of experimental design because they connect or not to our system *via* the Internet. However, the general characteristics of participants at baseline did not differ between ICT group and no-ICT group. Second, the reason for dropout could not be obtained from all the dropouts. This study was an RCT using ICT with a follow-up participation rate of more than 70% over 2 years and there was no difference in the baseline values of the main variables between dropouts and completers. This study is scheduled to be continuously tracked and still expects a high follow-up rate. Finally, there may be possible confounding factors between no-ICT group and ICT group that we have not yet considered. In addition, within the ICT group, the utilization of the web information system may differ by the ability to use the Internet. It is possible that changes in the utilization of the Internet will affect other lifestyles. However, we checked the internet-enabled environment and the ability of the participants to use the Internet before the intervention, and our web-based information system has a user-friendly user interface to make it easy for anyone to use. Moreover, we have evaluated the participants´ characteristics such as marital status, education, PA, tobacco use, alcohol use, sleep time, sleeplessness, and screen time. There were no differences between the ICT group and the no-ICT group at baseline and follow-up (Supplementary Table 1).

## Conclusions

This study demonstrates that HBCSS developed in our previous study [19] is able to reduce the prevalence of MetS during the 2-year follow-up period whereas non-ICT counselling methods had no effect. A clear dose–response effect was observed – the higher the utilization of the web information system, the lower the prevalence of MetS. Therefore, it can be concluded that this web-based ICT software helps patients with MetS to change and maintain their improved lifestyle in clinical practice.

## Supplementary Material

Supplemental Material
