# Supplementary material for: Lifestyle counselling by persuasive information and communications technology reduces prevalence of metabolic syndrome in a dose–response manner: a randomized clinical trial (PrevMetSyn)
Source: Ann Med. 2020 Jul 30;52(6):321–30. doi: 10.1080/07853890.2020.1783455 (PMC7877935; doi:10.1080/07853890.2020.1783455)
Supplement: Supplemental Material [file IANN_A_1783455_SM5677.docx]

**Supplementary Figure 1.** The flow chart of the participants in the study

**Supplementary Figure 1.** The flow chart of the participants in the study

**Supplementary Figure 1.** The flow chart of the participants in the study

Figure legend

***Figure legend.*** *The vertical bars show the visits to the study centre. There were three different conventional counselling methods (intensive group counselling, 8 times after the baseline visit; short counselling, 2 times after the baseline visit; no group counselling, a booklet with information and recommendations about healthy lifestyle). The higher vertical bars show the actual visits to the study centre at baseline, 12 months and 24 months. It is noteworthy that all study subjects had the same number of visits to the study centre and no other contacts with the study personnel. It is important to note that there were no intervention procedures between the 12-month and the 24-month visits.*

**Supplementary Figure 2.**


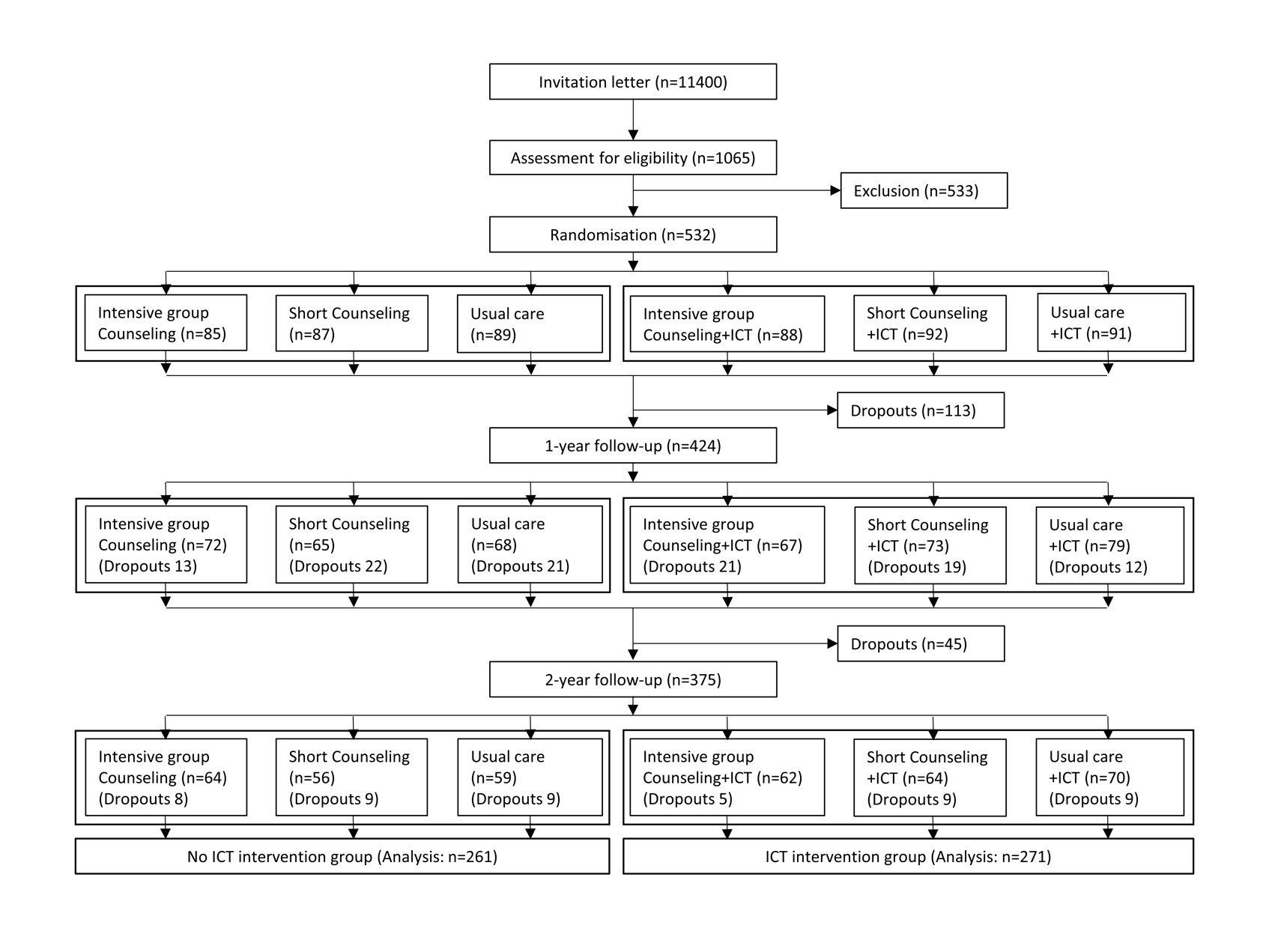


ICT, Information and Communication Technology.

**Supplementary Table 1.** General characteristics of no-ICT group and ICT group over time

|  | Baseline | | | 1-year follow-up | | | 2-year follow-up | | |
| --- | --- | --- | --- | --- | --- | --- | --- | --- | --- |
| Variables | No ICT  (n = 179) | ICT  (n = 196) | P value | No ICT  (n = 179) | ICT  (n = 196) | P value | No ICT  (n = 179) | ICT  (n = 196) | P value |
| Current Tobacco use, n (%) | 26 (16.2) | 42 (23.0) | 0.114 | 23 (16.3) | 28 (19.6) | 0.473 | 25 (18.7) | 24 (16.7) | 0.663 |
| Current Alcohol use, n (%) | 137 (85.1) | 162 (88.5) | 0.346 | 123 (87.2) | 129 (90.2) | 0.428 | 115 (85.8) | 129 (89.6) | 0.339 |
| Current Total PA, n (%) | 143 (89.4) | 162 (87.1) | 0.513 | 121 (90.3) | 127 (92.0) | 0.615 | 108 (88.5) | 122 (93.9) | 0.135 |
| Current Aerobic PA, n (%) | 143 (89.4) | 161 (86.6) | 0.424 | 121 (90.3) | 127 (92.0) | 0.615 | 108 (88.5) | 122 (93.9) | 0.135 |
| Current Resistant PA, n (%) | 49 (30.6) | 52 (28.0) | 0.586 | 55 (41.0) | 49 (35.5) | 0.347 | 55 (45.1) | 45 (34.6) | 0.090 |
| Sleep time, n (%) |  |  | 0.563 |  |  | 0.971 |  |  | 0.688 |
| <8 hours/day | 143 (88.8) | 166 (90.7) |  | 129 (91.5) | 131 (91.6) |  | 119 (88.8) | 130 (90.3) |  |
| ≥8 hours/day | 18 (11.2) | 17 (9.3) |  | 12 (8.5) | 12 (8.4) |  | 15 (11.2) | 14 (9.7) |  |
| Screen time, hours | 2.6±1.4 | 2.7±1.4 | 0.286 | 2.6±1.4 | 2.5±1.3 | 0.584 | 2.5±1.4 | 2.7±1.5 | 0.439 |
| Screen time, n (%) |  |  | 0.131 |  |  | 0.193 |  |  | 0.341 |
| <3 hours/day | 90 (56.3) | 88 (48.1) |  | 73 (51.8) | 85 (59.4) |  | 82 (61.2) | 80 (55.6) |  |
| ≥3 hours/day | 70 (43.8) | 95 (51.9) |  | 68 (48.2) | 58 (40.6) |  | 52 (38.8) | 64 (44.4) |  |
| Eating while Watching, n (%) | 126 (78.8) | 153 (83.6) | 0.249 | 110 (78.0) | 118 (82.5) | 0.340 | 102 (76.1) | 123 (85.4) | 0.049 |
| Current Stress, n (%) | 130 (80.8) | 154 (84.2) | 0.406 | 114 (80.9) | 112 (78.3) | 0.597 | 103 (76.9) | 109 (75.7) | 0.819 |

ICT, Information and Communication Technology; PA, Physical Activity.

Data are presented as mean±standard deviation for continuous variables (T test) and numbers (%) for categorical variables (χ^2^ test). Percentages have been rounded and may not total 100.

**Supplementary Table 2.**Effectors for Metabolic Syndrome between no-ICT and ICT groups

| Variables | Mixed Model1  OR (95%CI) | GEE Model1  OR (95%CI) | Mixed Model2  OR (95%CI) | GEE Model2  OR (95%CI) |
| --- | --- | --- | --- | --- |
| Age | 1.15 (1.11 to 1.20) | 1.08 (1.06 to 1.11) | 1.16 (1.11 to 1.21) | 1.09 (1.06 to 1.11) |
| Sex |  |  |  |  |
| Men | reference | reference | reference | reference |
| Women | 0.39 (0.21 to 0.73) | 0.58 (0.41 to 0.82) | 0.35 (0.18 to 0.67) | 0.55 (0.39 to 0.78) |
| Group |  |  |  |  |
| No-ICT intervention | reference | reference | reference | reference |
| ICT intervention | 0.50 (0.27 to 0.90) | 0.67 (0.48 to 0.93) | 0.50 (0.27 to 0.93) | 0.67 (0.48 to 0.94) |
| Time |  |  |  |  |
| Baseline | reference | reference | reference | reference |
| 1-year follow-up | 0.40 (0.25 to 0.64) | 0.59 (0.46 to 0.77) | 0.38 (0.23 to 0.62) | 0.58 (0.44 to 0.76) |
| 2-year follow-up | 0.61 (0.38 to 0.99) | 0.75 (0.58 to 0.98) | 0.68 (0.41 to 1.12) | 0.79 (0.60 to 1.04) |
| Obesity |  |  |  |  |
| BMI <30 kg/m^2^ | reference | reference | reference | reference |
| BMI ≥30 kg/m^2^ | 4.99 (2.86 to 8.71) | 2.43 (1.81 to 3.27) | 5.04 (2.81 to 9.02) | 2.44 (1.79 to 3.31) |
| Current Tobacco use |  |  |  |  |
| No | reference | reference | reference | reference |
| Yes | 2.28 (1.12 to 4.61) | 1.64 (1.09 to 2.46) | 2.63 (1.25 to 5.54) | 1.76 (1.17 to 2.65) |
| Eating while Watching |  |  |  |  |
| No | reference | reference | reference | reference |
| Yes | 2.66 (1.38 to 5.14) | 1.81 (1.22 to 2.67) | 3.18 (1.59 to 6.35) | 1.97 (1.31 to 2.95) |

ICT, Information and Communication Technology; OR, odds ratio; CI, confidence interval; BMI, Body Mass Index; PA, Physical Activity.

Mixed Model: mixed effects logistic regression model (random effect: individual)

GEE Model: generalised estimating equations model.

Model 1: adjusted for age, sex, time, obesity, current tobacco use, eating while watching, and type of group counselling (no, short-term, or intensive).

Model 2: model 1 + sleep time and current resistant physical activity.

**Supplementary Table 3.**Effect of degree of utilisation of internet-based lifestyle counselling on the prevalence of Metabolic Syndrome

| Variables | Mixed Model1  OR (95%CI) | P value | GEE Model1  OR (95%CI) | P value |
| --- | --- | --- | --- | --- |
| Number of Logins |  |  |  |  |
| No ICT | reference |  | reference |  |
| 1st tertile | 1.01 (0.43 to 2.41) | 0.975 | 0.98 (0.60 to 1.60) | 0.932 |
| 2nd tertile | 0.45 (0.19 to 1.04) | 0.060 | 0.64 (0.39 to 1.03) | 0.064 |
| 3rd tertile | 0.29 (0.12 to 0.68) | 0.004 | 0.49 (0.31 to 0.76) | 0.002 |
| Number of Weekly task records |  |  |  |  |
| No ICT | reference |  | reference |  |
| 1st tertile | 0.79 (0.37 to 1.67) | 0.533 | 0.86 (0.56 to 1.31) | 0.474 |
| 2nd tertile | 0.39 (0.14 to 1.09) | 0.074 | 0.58 (0.33 to 1.03) | 0.064 |
| 3rd tertile | 0.30 (0.13 to 0.71) | 0.006 | 0.52 (0.33 to 0.83) | 0.006 |
| Number of Body weight records |  |  |  |  |
| No ICT | reference |  | reference |  |
| 1st tertile | 1.00 (0.44 to 2.27) | 0.999 | 0.97 (0.61 to 1.56) | 0.910 |
| 2nd tertile | 0.37 (0.15 to 0.89) | 0.026 | 0.56 (0.35 to 0.91) | 0.020 |
| 3rd tertile | 0.32 (0.14 to 0.73) | 0.007 | 0.52 (0.33 to 0.83) | 0.005 |
| Number of Diary records |  |  |  |  |
| No ICT | reference |  | reference |  |
| 1st tertile | 0.72 (0.35 to 1.49) | 0.381 | 0.81 (0.54 to 1.22) | 0.309 |
| 2nd tertile | 0.30 (0.09 to 0.96) | 0.043 | 0.52 (0.29 to 0.96) | 0.037 |
| 3rd tertile | 0.35 (0.15 to 0.82) | 0.015 | 0.55 (0.34 to 0.88) | 0.013 |
| Number of Exercise records |  |  |  |  |
| No ICT | reference |  | reference |  |
| 1st tertile | 0.99 (0.46 to 2.15) | 0.988 | 0.98 (0.64 to 1.52) | 0.944 |
| 2nd tertile | 0.30 (0.12 to 0.77) | 0.013 | 0.50 (0.31 to 0.83) | 0.007 |
| 3rd tertile | 0.32 (0.14 to 0.73) | 0.007 | 0.50 (0.31 to 0.81) | 0.005 |
| Number of Food diary records |  |  |  |  |
| No ICT | reference |  | reference |  |
| 1st tertile | 0.74 (0.35 to 1.55) | 0.423 | 0.82 (0.54 to 1.23) | 0.332 |
| 2nd tertile | 0.83 (0.28 to 2.45) | 0.738 | 0.94 (0.52 to 1.69) | 0.833 |
| 3rd tertile | 0.22 (0.09 to 0.51) | 0.001 | 0.42 (0.26 to 0.68) | < 0.001 |

ICT, Information and Communication Technology; OR, odds ratio; CI, confidence interval.

Mixed Model: mixed effects logistic regression model (random effect: individual).

GEE Model: generalised estimating equations model.

Model 1: adjusted for age, sex, time, obesity, current tobacco use, eating while watching, and type of group counselling (no, short-term, or intensive).

**Supplementary Table 4.**Mixed Effects Linear Regression models for each outcome between no-ICT group and ICT group

| Variables | No ICT(n = 261)  mean±standard deviation | ICT(n = 271)  mean±standard deviation | β | 95%CI | P value |
| --- | --- | --- | --- | --- | --- |
| Body weight (kg) |  |  |  |  |  |
| Baseline | 89.7±11.6 | 89.3±10.9 |  |  |  |
| 1-year follow-up | 88.6±11.8 | 87.1±12.0 | -1.50 | -2.37 to -0.63 | 0.001 |
| 2-year follow-up | 89.1±11.8 | 87.3±12.2 | -1.32 | -2.23 to -0.41 | 0.005 |
| BMI (kg/m^2^) |  |  |  |  |  |
| Baseline | 30.6±2.1 | 30.3±2.1 |  |  |  |
| 1-year follow-up | 30.2±2.5 | 29.4±2.3 | -0.54 | -0.83 to -0.24 | <0.001 |
| 2-year follow-up | 30.3±2.6 | 29.5±2.3 | -0.50 | -0.81 to -0.19 | 0.002 |
| WC (cm) |  |  |  |  |  |
| Baseline | 101.1±7.6 | 100.8±8.1 |  |  |  |
| 1-year follow-up | 100.4±8.0 | 98.2±8.8 | -1.78 | -2.68 to -0.88 | <0.001 |
| 2-year follow-up | 99.9±8.4 | 97.5±8.9 | -1.61 | -2.55 to -0.68 | 0.001 |
| SBP (mmHg) |  |  |  |  |  |
| Baseline | 130.6±16.8 | 130.0±15.8 |  |  |  |
| 1-year follow-up | 124.8±16.9 | 124.6±16.7 | 0.73 | -1.99 to 3.45 | 0.600 |
| 2-year follow-up | 127.5±17.3 | 124.6±14.5 | -1.47 | -4.32 to 1.37 | 0.310 |
| DBP (mmHg) |  |  |  |  |  |
| Baseline | 82.7±10.5 | 82.5±10.0 |  |  |  |
| 1-year follow-up | 79.0±10.5 | 78.5±10.0 | -0.20 | -1.70 to 1.30 | 0.793 |
| 2-year follow-up | 80.9±11.2 | 79.5±9.7 | -0.83 | -2.39 to 0.74 | 0.301 |
| HbA1c (%) |  |  |  |  |  |
| Baseline | 5.68±0.40 | 5.69±0.44 |  |  |  |
| 1-year follow-up | 5.58±0.35 | 5.64±0.49 | 0.04 | -0.02 to 0.09 | 0.166 |
| 2-year follow-up | 5.49±0.34 | 5.49±0.48 | -0.02 | -0.08 to 0.04 | 0.557 |
| FPG (mmol/L) |  |  |  |  |  |
| Baseline | 5.52±0.47 | 5.52±0.59 |  |  |  |
| 1-year follow-up | 5.51±0.49 | 5.49±0.62 | -0.03 | -0.13 to 0.07 | 0.547 |
| 2-year follow-up | 5.51±0.57 | 5.42±0.66 | -0.10 | -0.21 to 0.004 | 0.059 |
| TC (mmol/L) |  |  |  |  |  |
| Baseline | 5.33±0.98 | 5.40±0.99 |  |  |  |
| 1-year follow-up | 5.27±1.00 | 5.28±1.07 | -0.07 | -0.22 to 0.07 | 0.325 |
| 2-year follow-up | 5.16±0.94 | 5.33±1.01 | 0.06 | -0.09 to 0.21 | 0.460 |
| HDL (mmol/L) |  |  |  |  |  |
| Baseline | 1.45±0.33 | 1.48±0.39 |  |  |  |
| 1-year follow-up | 1.42±0.32 | 1.48±0.42 | 0.03 | -0.02 to 0.07 | 0.235 |
| 2-year follow-up | 1.45±0.38 | 1.52±0.44 | 0.04 | -0.01 to 0.08 | 0.105 |
| LDL (mmol/L) |  |  |  |  |  |
| Baseline | 3.61±0.91 | 3.64±0.99 |  |  |  |
| 1-year follow-up | 3.59±0.94 | 3.44±0.97 | -0.18 | -0.31 to -0.04 | 0.010 |
| 2-year follow-up | 3.52±0.89 | 3.61±0.97 | 0.03 | -0.11 to 0.17 | 0.695 |
| TG (mmol/L)^*^ |  |  |  |  |  |
| Baseline | 1.26±1.67 | 1.30±1.62 |  |  |  |
| 1-year follow-up | 1.14±1.74 | 1.18±1.62 | -1.01 | -1.09 to 1.06 | 0.755 |
| 2-year follow-up | 1.17±1.61 | 1.21±1.60 | -1.02 | -1.10 to 1.06 | 0.694 |
| Insulin (mIU/L) |  |  |  |  |  |
| Baseline | 13.3±6.4 | 12.4±5.6 |  |  |  |
| 1-year follow-up | 13.1±7.3 | 11.3±5.9 | -0.60 | -1.53 to 0.34 | 0.212 |
| 2-year follow-up | 13.5±7.8 | 12.0±6.1 | -0.35 | -1.33 to 0.62 | 0.479 |

ICT, Information and Communication Technology; CI, confidence interval;BMI, Body Mass Index; WC, Waist Circumference; SBP, Systolic Blood Pressure; DBP, Diastolic Blood Pressure; HbA1c, Haemoglobin A1c; FPG, Fasting Plasma Glucose; TC, Total Cholesterol; HDL, High-Density Lipoprotein; LDL, Low-Density Lipoprotein; TG, Triglycerides.

β: group × time interaction effects adjusted for age and sex in the mixed effects linear regression models.

^*^Geometric mean±standard deviation.
